# Crystal structure of the chalcone (*E*)-3-(furan-2-yl)-1-phenylprop-2-en-1-one

**DOI:** 10.1107/S205698901500047X

**Published:** 2015-01-17

**Authors:** Oscar F. Vázquez-Vuelvas, René A. Enríquez-Figueroa, Héctor García-Ortega, Marcos Flores-Alamo, Armando Pineda-Contreras

**Affiliations:** a Facultad de Ciencias Químicas, Universidad de Colima, Km 9 Carr. Colima-Coquimatlán s/n, Coquimatlán, Colima 28400, Mexico; b Facultad de Química, Universidad Nacional Autónoma de México, Ciudad Universitaria, México, DF 04510, Mexico

**Keywords:** crystal structure, Claisen–Schmidt reaction, chalcone derivative, hydrogen bonding, biological activity, zigzag fashion.

## Abstract

The title chalcone derivative, C_13_H_10_O_2_, adopts an *E* conformation about the C=C double bond. The mol­ecule is composed of a furanyl and a phenyl ring, bridged by an α,β-unsaturated carbonyl system, which are inclined to one another by 24.07 (7)°. In the crystal, mol­ecules are connected by weak C—H⋯O hydrogen bonds involving the carbonyl O atom acting as a trifurcated acceptor and C—H⋯π inter­actions, forming ribbons extending along the *c-*axis direction.

## Abstract 2

The crystal packing of the compound is described by an inter­molecular arrangement with the mol­ecules as inter­laced layers in a zigzag fashion, denoting inter­acting self-complementary dimers mainly by the localization of weak hydrogen bonds in a head-to-tail arrangement.

## Chemical context   

The Claisen–Schmidt condensation reaction between substituted aceto­phenones and aryl aldehydes under basic con­ditions has been widely used to synthesize chalcone derivatives (Ghosh & Das, 2014 ▸; Robinson *et al.*, 2013 ▸; Sharma *et al.*, 2013 ▸; Tiwari *et al.*, 2010 ▸). Chalcones, belonging to the flavonoid family, are an important class of natural products with widespread distribution in fruits, vegetables, spices and tea. These compounds are also often used as the precursors for the synthesis of various heterocyclic compounds (Chimenti *et al.*, 2010 ▸; Elarfi & Al-Difar, 2012 ▸; Ghosh & Das, 2014 ▸; Hamada & Sharshira, 2011 ▸; Mahé *et al.*, 2012 ▸; Sharma *et al.*, 2013 ▸). Chemically, chalcones are 1,3-diaryl-2-propen-1-ones in which two aromatic rings, mainly benzene groups, are joined by a three-carbon bridge having a carbonyl moiety and α,β-unsaturation.

 Many studies have shown that chalcone derivatives exhibit a wide range of pharmacological activities, such as potential cytotoxic, anti­microbial, anti­viral, anti-inflammatory, anti-oxidant, anaesthetic, anti­malarial, anti­leishmanial, anti­tubercular, anti­tumor and anti­cancer activities (Boeck *et al.*, 2006 ▸; Chimenti *et al.*, 2010 ▸; Elarfi & Al-Difar, 2012 ▸; Hamada & Sharshira, 2011 ▸; Hsieh *et al.*, 2000 ▸; Kumar *et al.*, 2003 ▸; Sharma *et al.*, 2013 ▸). These versatile compounds and their furan deriv­atives are often used as inter­mediates in the syntheses of mono­amine oxidase (MAO) inhibitors; moreover, the chalcones themselves have MAO inhibitory activity. Since the furan moiety represents a high π-electron density that contributes to the inter­action with the flavin nucleus of the co-factor in the inhibition of MAO, some furan-substituted chalcones, where an electron-rich heterocyclic oxygen replaces the benzene ring, have been synthesized to investigate their biological activity (Robinson *et al.*, 2013 ▸; Shaikh *et al.*, 2014 ▸; Sharma *et al.*, 2013 ▸; Zheng *et al.*, 2011 ▸). In view of the varied biological and pharmacological applications, we report herein on the synthesis and the mol­ecular and supra­molecular structure of the title compound, synthesized by a conventional base-catalysed Claisen–Schmidt condensation reaction.
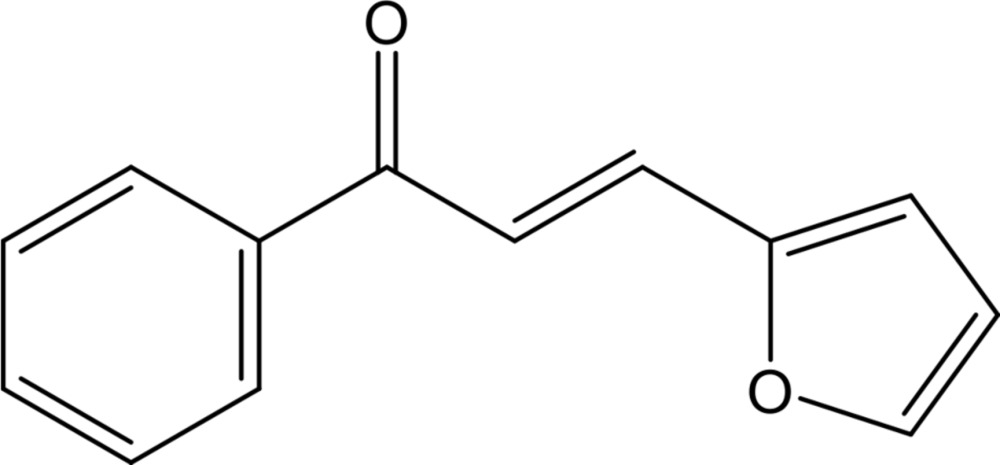


## Structural commentary   

**Figure 1:**
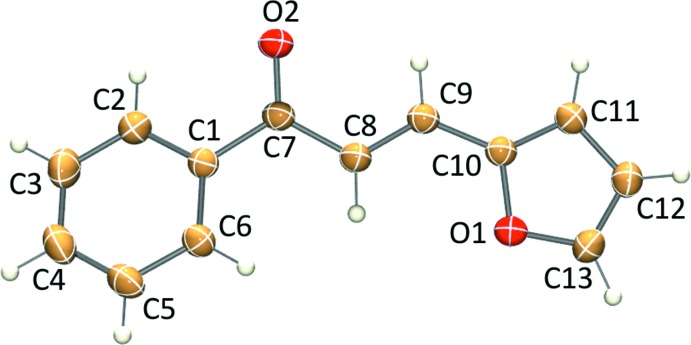
The mol­ecular structure of the title compound, with displacement ellipsoids drawn at the 30% probability level.

The symmetry-independent mol­ecule adopts an *E* conformation corresponding to an α,β-unsaturated non-planar structure, which bridges the pair of aromatic groups (Fig. 1 ▸). The two main planar groups, the furanyl and the phenyl rings, form a dihedral angle of 24.07 (7)°. In this context, the mol­ecular structure can be considered, for descriptive purposes, as two fragments basically described by the furanyl acryloyl and the benzoyl moieties. The benzoyl group shows a non-planar structure and presents rotation when observing the C2—C1—C7—O2 torsion angle of 19.4 (2)°, denoting a marked deviation from planarity at the C1—C7 bond, a single bond with rotational freedom. This deviation from planarity has also been reported previously in the crystal structure of an (*E*)-3-(4-hy­droxy­phen­yl)-1-(4-meth­oxy­phen­yl)-prop-2-en-1-one derivative, when observing the analogous reported inter­planar angle shown in the respectively 4-meth­oxy­benzoyl moiety (Qiu *et al.*, 2006 ▸). In the same manner, the furanyl acryloyl entity presents a quasi-planar structure indicated by the two small torsion angles O2—C7—C8—C9 [−5.4 (2)°] and C7—C8—C9—C10 [−176.31 (13)°], similar to the structure of the di­furanyl chalcone derivative (*E)*-1,3-di(2-fur­yl)-2-propen-1-one (Ocak Iskeleli *et al.*, 2005*b* ▸). On the other hand, the mol­ecule inter­atomic linkage coincides with similar reported structures, specifically in the α,β-unsaturated entity of the title crystal (Harrison *et al.*, 2006 ▸; Ocak Iskeleli *et al.*, 2005*a* ▸,*b* ▸). As a result, the inter­atomic distances are in agreement with the conjugative nature, which is additionally supported by other described types of different weak inter­actions (*vide infra*) and also define the characteristic quasi-planar structure of chalcone derivatives.

## Supra­molecular features   

**Figure 2:**
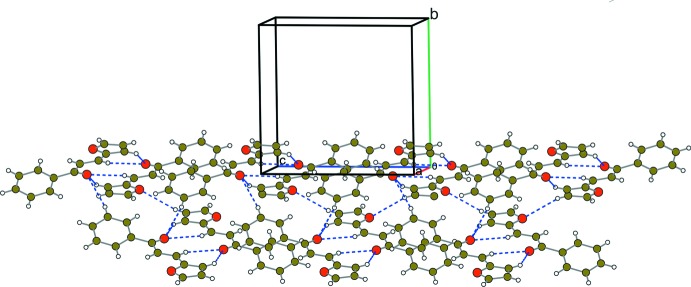
A partial packing diagram of the title compound, showing the hydrogen-bonded supra­molecular assembly *via* C—H⋯O inter­actions (blue dashed lines).

**Figure 3:**
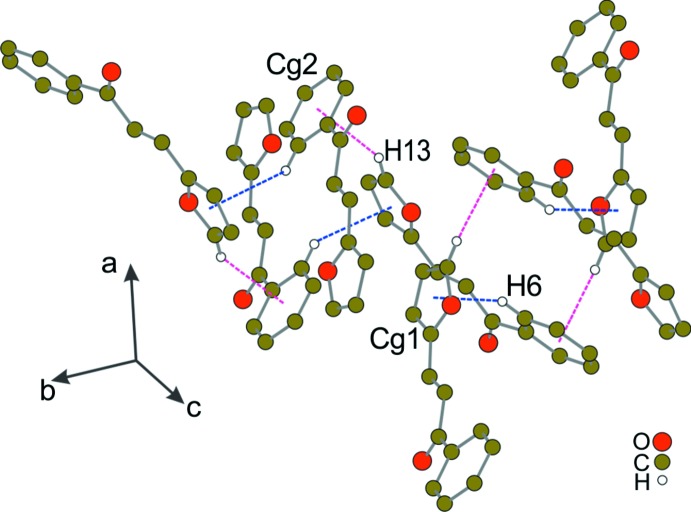
A partial packing diagram of the title compound, showing the C—H⋯π stacking inter­actions, depicted as blue and purple dotted lines for the C6—H6⋯*Cg*1 and C13—H13⋯*Cg*2 contacts, respectively. H atoms not involved in hydrogen-bonding inter­actions have been omitted for clarity.

The crystal packing does not present geometrical parameters corresponding to classical hydrogen bonding (Gilli & Gilli, 2009 ▸; Steiner, 2002 ▸), neither intra- nor inter­molecular. In the crystal, centrosymmetrically related mol­ecules inter­act through a pair of weak hydrogen contacts (Table 1 ▸) with the C9 and C11 carbon atoms as donors and the O2 oxygen atom as a bifurcated acceptor, generating a ring with an 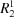
(6) graph-set motif (Bernstein *et al.*, 1995 ▸). The reciprocal inter­actions with the corresponding mol­ecule positioned in a head-to-tail mode generate the same ring motif and, as a consequence, an 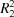
(10) ring is formed, describing a three-fused-ring system (Fig. 2 ▸). In addition, a weak hydrogen contact is present involving the C3 carbon atom as H-donor and the O2 oxygen atom acting, in this way, as a trifurcated acceptor. The propagation of this inter­action generates a ribbon along the *c-*axis direction (Fig. 2 ▸). The supra­molecular assembly is additionally supported by weak C—H⋯π inter­actions, implicating the phenyl and furanyl π systems (Fig. 3 ▸).

## Synthesis and crystallization   

To a solution of NaOH (2.18 g, 55 mmol) in H_2_O/EtOH (30 ml, 2:1 *v*/*v*) was added pure aceto­phenone (5.2 g, 43 mmol), and stirring started; furfuraldehyde (4.6 g, 43 mmol) was then added at once. The reaction mixture was stirred for two hours and then kept in a refrigerator overnight. The resulting product was separated and then distilled under vacuum. The title compound was obtained as a yellow solid in 82% yield. Single-crystals suitable for X-ray determination were obtained by evaporation of an ethyl ether solution. M.p. 311–313 K; IR (ν, cm^−1^): 3123 (C—H_alk_), 3035 (C—H_arom_), 1658 (C=O), 1594, 1545, 1474 (C=C). ^1^H NMR (400 MHz, CDCl_3_, δ, p.p.m.): 8.04 (2H, *dd*), 7.61 (1H, *d*), 7.58 (1H, *tt*), 7.52 (2H, *dd*), 7.49 (1H, *dd*), 7.47 (1H, *d*), 6.72 (1H, *dd*), 6.51 (1H, *dd*). ^13^C NMR (100 MHz, CDCl_3_, δ, p.p.m.): 189.85 (C7), 151.69 (C10), 144.98 (C13), 138.16 (C1), 132.82 (C4), 130.72 (C9), 128.65 (C2,6), 128.47 (C3,5), 119.30 (C8), 116.33 (C11), 112.74 (C12). MS *m*/*z*: 199 (*M* +1); Analysis calculated for C_13_H_10_O_2_: C, 78.78; H, 5.05. Found: 78.80; H, 5.09.

## Refinement details   

Crystal data, data collection and structure refinement details are summarized in Table 2 ▸. H atoms attached to C atoms were placed in geometrically idealized positions and refined as riding on their parent atoms, with C—H = 0.95 Å and with *U_iso_*(H) = 1.2*U_eq_*(C).

## Supplementary Material

Crystal structure: contains datablock(s) global, I. DOI: 10.1107/S205698901500047X/rz5145sup1.cif

Structure factors: contains datablock(s) I. DOI: 10.1107/S205698901500047X/rz5145Isup2.hkl

Supporting information file. DOI: 10.1107/S205698901500047X/rz5145Isup3.cml

CCDC reference: 1042952

Additional supporting information:  crystallographic information; 3D view; checkCIF report

## Figures and Tables

**Table 1:** Hydrogen-bond geometry (, ) *Cg*1 and *Cg*2 are the centroids of the O1/C10C13 furanyl ring and the C1C6 phenyl ring, respectively.

*D*H*A*	*D*H	H*A*	*D* *A*	*D*H*A*
C3H3O2^i^	0.95	2.51	3.457(2)	151
C9H9O2^ii^	0.95	2.62	3.416(1)	142
C11H11O2^ii^	0.95	2.51	3.277(2)	138
C6H6*Cg*1^iii^	0.95	2.88	3.687(2)	144
C13H13*Cg*2^iv^	0.95	2.71	3.519(9)	143

Symmetry codes: (i) 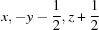; (ii) 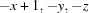; (iii) 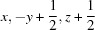; (iv) 
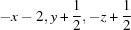
.

**Table 2:** Experimental details

Crystal data	
Chemical formula	C_13_H_10_O_2_
*M*_r_	198.21
Crystal system, space group	Monoclinic, *P*2_1_/*c*
Temperature (K)	130
*a*, *b*, *c* ()	9.5296(7), 10.1383(7), 11.1595(7)
()	103.922(6)
*V* (^3^)	1046.49(13)
*Z*	4
Radiation type	Mo *K*
(mm^1^)	0.08
Crystal size (mm)	0.50 0.45 0.32

Data collection	
Diffractometer	Agilent Xcalibur Atlas Gemini
Absorption correction	Multi-scan (*CrysAlis PRO*; Agilent, 2011 ▸)
*T*_min_, *T*_max_	0.781, 1
No. of measured, independent and observed [*I* > 2(*I*)] reflections	8114, 2553, 1755
*R*_int_	0.019
(sin /)_max_ (^1^)	0.691

Refinement	
*R*[*F* ^2^ > 2(*F* ^2^)], *wR*(*F* ^2^), *S*	0.045, 0.115, 1.04
No. of reflections	2553
No. of parameters	136
H-atom treatment	H-atom parameters constrained
_max_, _min_ (e ^3^)	0.11, 0.17

Computer programs: *CrysAlis PRO* (Agilent, 2011 ▸), *SHELXS2013* and *SHELXL2013* (Sheldrick, 2008 ▸, 2015 ▸), *ORTEP-3 for Windows* and *WinGX* (Farrugia, 2012 ▸), *Mercury* (Macrae *et al.*, 2006 ▸) and *DIAMOND* (Brandenburg, 2012 ▸).

## supplementary crystallographic information

### Crystal data

**Table:**

C_13_H_10_O_2_	*F*(000) = 416
*M**_r_* = 198.21	*D*_x_ = 1.258 Mg m^−^^3^
Monoclinic, *P*2_1_/*c*	Mo *K*α radiation, λ = 0.71073 Å
Hall symbol: -P 2ybc	Cell parameters from 2252 reflections
*a* = 9.5296 (7) Å	θ = 3.8–29.4°
*b* = 10.1383 (7) Å	µ = 0.08 mm^−^^1^
*c* = 11.1595 (7) Å	*T* = 130 K
β = 103.922 (6)°	Prism, colorless
*V* = 1046.49 (13) Å^3^	0.50 × 0.45 × 0.32 mm
*Z* = 4	

### Data collection

**Table:**

Agilent Xcalibur Atlas Gemini diffractometer	2553 independent reflections
Graphite monochromator	1755 reflections with *I* > 2σ(*I*)
Detector resolution: 10.4685 pixels mm^-1^	*R*_int_ = 0.019
ω scans	θ_max_ = 29.4°, θ_min_ = 3.8°
Absorption correction: multi-scan (*CrysAlis PRO*; Agilent, 2011)	*h* = −12→11
*T*_min_ = 0.781, *T*_max_ = 1	*k* = −13→14
8114 measured reflections	*l* = −14→14

### Refinement

**Table:**

Refinement on *F*^2^	0 restraints
Least-squares matrix: full	Hydrogen site location: inferred from neighbouring sites
*R*[*F*^2^ > 2σ(*F*^2^)] = 0.045	H-atom parameters constrained
*wR*(*F*^2^) = 0.115	*w* = 1/[σ^2^(*F*_o_^2^) + (0.0462*P*)^2^ + 0.1328*P*] where *P* = (*F*_o_^2^ + 2*F*_c_^2^)/3
*S* = 1.04	(Δ/σ)_max_ < 0.001
2553 reflections	Δρ_max_ = 0.11 e Å^−^^3^
136 parameters	Δρ_min_ = −0.17 e Å^−^^3^

### Special details

**Table:**

Experimental. Absorption correction: CrysAlisPro, Agilent Technologies (Agilent, 2011) Empirical absorption correction using spherical harmonics, implemented in SCALE3 ABSPACK scaling algorithm.
Geometry. All e.s.d.'s (except the e.s.d. in the dihedral angle between two l.s. planes) are estimated using the full covariance matrix. The cell e.s.d.'s are taken into account individually in the estimation of e.s.d.'s in distances, angles and torsion angles; correlations between e.s.d.'s in cell parameters are only used when they are defined by crystal symmetry. An approximate (isotropic) treatment of cell e.s.d.'s is used for estimating e.s.d.'s involving l.s. planes.

### Fractional atomic coordinates and isotropic or equivalent isotropic displacement parameters (Å^2^)

**Table:**

	*x*	*y*	*z*	*U*_iso_*/*U*_eq_	
O1	0.96039 (10)	0.15914 (10)	0.09922 (8)	0.0619 (3)	
C1	0.65936 (13)	−0.05129 (13)	0.39220 (11)	0.0465 (3)	
O2	0.50969 (10)	−0.03589 (12)	0.19207 (8)	0.0724 (3)	
C7	0.62831 (14)	−0.01269 (13)	0.25976 (11)	0.0499 (3)	
C9	0.72460 (14)	0.06998 (13)	0.09060 (12)	0.0498 (3)	
H9	0.6322	0.049	0.0396	0.06*	
C8	0.74268 (14)	0.04814 (14)	0.21095 (11)	0.0516 (3)	
H8	0.8312	0.0724	0.2662	0.062*	
C10	0.82803 (14)	0.12119 (13)	0.02963 (11)	0.0477 (3)	
C11	0.82469 (15)	0.13668 (14)	−0.09089 (12)	0.0549 (3)	
H11	0.7452	0.1182	−0.1585	0.066*	
C6	0.76975 (15)	0.00494 (14)	0.48148 (12)	0.0551 (4)	
H6	0.8309	0.0694	0.4587	0.066*	
C12	0.96066 (16)	0.18535 (14)	−0.09817 (13)	0.0587 (4)	
H12	0.991	0.2057	−0.1711	0.07*	
C2	0.57275 (16)	−0.14617 (15)	0.42759 (13)	0.0605 (4)	
H2	0.4966	−0.1857	0.3675	0.073*	
C13	1.03802 (17)	0.19718 (16)	0.01731 (14)	0.0649 (4)	
H13	1.1349	0.2281	0.0399	0.078*	
C5	0.79115 (17)	−0.03262 (17)	0.60379 (13)	0.0674 (4)	
H5	0.8662	0.0069	0.6649	0.081*	
C4	0.70484 (19)	−0.12620 (19)	0.63691 (15)	0.0745 (5)	
H4	0.72	−0.1517	0.721	0.089*	
C3	0.59661 (19)	−0.18335 (18)	0.54951 (16)	0.0740 (5)	
H3	0.5375	−0.2492	0.573	0.089*	

### Atomic displacement parameters (Å^2^)

**Table:**

	*U*^11^	*U*^22^	*U*^33^	*U*^12^	*U*^13^	*U*^23^
O1	0.0544 (6)	0.0759 (7)	0.0512 (5)	−0.0135 (5)	0.0044 (4)	0.0031 (5)
C1	0.0452 (7)	0.0491 (7)	0.0454 (7)	0.0025 (5)	0.0115 (5)	−0.0024 (5)
O2	0.0504 (6)	0.1151 (9)	0.0482 (6)	−0.0173 (6)	0.0049 (4)	−0.0038 (6)
C7	0.0457 (7)	0.0577 (8)	0.0448 (7)	−0.0021 (6)	0.0080 (5)	−0.0071 (6)
C9	0.0469 (7)	0.0538 (8)	0.0471 (7)	−0.0012 (6)	0.0085 (5)	−0.0031 (6)
C8	0.0476 (7)	0.0602 (8)	0.0455 (7)	−0.0060 (6)	0.0081 (5)	−0.0030 (6)
C10	0.0470 (7)	0.0475 (7)	0.0468 (7)	−0.0015 (5)	0.0078 (5)	−0.0010 (5)
C11	0.0556 (8)	0.0610 (8)	0.0473 (7)	−0.0025 (7)	0.0110 (6)	−0.0014 (6)
C6	0.0585 (8)	0.0595 (8)	0.0467 (7)	−0.0017 (6)	0.0113 (6)	−0.0054 (6)
C12	0.0610 (9)	0.0609 (9)	0.0582 (8)	0.0001 (7)	0.0217 (7)	0.0050 (7)
C2	0.0546 (8)	0.0661 (9)	0.0605 (9)	−0.0038 (7)	0.0129 (6)	0.0034 (7)
C13	0.0533 (8)	0.0721 (10)	0.0702 (10)	−0.0104 (7)	0.0164 (7)	0.0086 (8)
C5	0.0675 (10)	0.0847 (11)	0.0461 (8)	0.0074 (8)	0.0060 (7)	−0.0061 (8)
C4	0.0788 (11)	0.0943 (13)	0.0523 (9)	0.0182 (10)	0.0193 (8)	0.0168 (9)
C3	0.0742 (11)	0.0777 (11)	0.0742 (11)	0.0007 (8)	0.0256 (9)	0.0199 (9)

### Geometric parameters (Å, º)

**Table:**

O1—C13	1.3627 (17)	C11—H11	0.95
O1—C10	1.3678 (15)	C6—C5	1.3840 (19)
C1—C2	1.3855 (19)	C6—H6	0.95
C1—C6	1.3857 (18)	C12—C13	1.327 (2)
C1—C7	1.4882 (17)	C12—H12	0.95
O2—C7	1.2218 (15)	C2—C3	1.377 (2)
C7—C8	1.4664 (18)	C2—H2	0.95
C9—C8	1.3308 (17)	C13—H13	0.95
C9—C10	1.4236 (18)	C5—C4	1.364 (2)
C9—H9	0.95	C5—H5	0.95
C8—H8	0.95	C4—C3	1.366 (2)
C10—C11	1.3468 (18)	C4—H4	0.95
C11—C12	1.407 (2)	C3—H3	0.95
			
C13—O1—C10	105.91 (10)	C5—C6—H6	119.9
C2—C1—C6	118.81 (12)	C1—C6—H6	119.9
C2—C1—C7	118.43 (11)	C13—C12—C11	106.25 (13)
C6—C1—C7	122.76 (12)	C13—C12—H12	126.9
O2—C7—C8	120.74 (12)	C11—C12—H12	126.9
O2—C7—C1	119.78 (12)	C3—C2—C1	120.25 (14)
C8—C7—C1	119.44 (11)	C3—C2—H2	119.9
C8—C9—C10	127.34 (12)	C1—C2—H2	119.9
C8—C9—H9	116.3	C12—C13—O1	111.18 (13)
C10—C9—H9	116.3	C12—C13—H13	124.4
C9—C8—C7	121.16 (12)	O1—C13—H13	124.4
C9—C8—H8	119.4	C4—C5—C6	120.22 (15)
C7—C8—H8	119.4	C4—C5—H5	119.9
C11—C10—O1	109.34 (12)	C6—C5—H5	119.9
C11—C10—C9	131.84 (12)	C5—C4—C3	120.17 (14)
O1—C10—C9	118.76 (11)	C5—C4—H4	119.9
C10—C11—C12	107.32 (12)	C3—C4—H4	119.9
C10—C11—H11	126.3	C4—C3—C2	120.40 (16)
C12—C11—H11	126.3	C4—C3—H3	119.8
C5—C6—C1	120.15 (14)	C2—C3—H3	119.8
			
C2—C1—C7—O2	19.4 (2)	C9—C10—C11—C12	−176.65 (14)
C6—C1—C7—O2	−159.66 (14)	C2—C1—C6—C5	−0.7 (2)
C2—C1—C7—C8	−158.15 (13)	C7—C1—C6—C5	178.37 (13)
C6—C1—C7—C8	22.74 (19)	C10—C11—C12—C13	−0.29 (17)
C10—C9—C8—C7	−176.31 (13)	C6—C1—C2—C3	0.0 (2)
O2—C7—C8—C9	−5.4 (2)	C7—C1—C2—C3	−179.11 (14)
C1—C7—C8—C9	172.14 (12)	C11—C12—C13—O1	−0.03 (18)
C13—O1—C10—C11	−0.50 (16)	C10—O1—C13—C12	0.33 (17)
C13—O1—C10—C9	177.07 (12)	C1—C6—C5—C4	0.8 (2)
C8—C9—C10—C11	173.72 (14)	C6—C5—C4—C3	−0.1 (2)
C8—C9—C10—O1	−3.2 (2)	C5—C4—C3—C2	−0.7 (3)
O1—C10—C11—C12	0.49 (16)	C1—C2—C3—C4	0.7 (3)

### Hydrogen-bond geometry (Å, º)

Cg1 and Cg2 are the centroids of the O1/C10–C13 furanyl ring and the C1–C6 phenyl ring, respectively.

**Table:**

*D*—H···*A*	*D*—H	H···*A*	*D*···*A*	*D*—H···*A*
C3—H3···O2^i^	0.95	2.51	3.457 (2)	151
C9—H9···O2^ii^	0.95	2.62	3.416 (1)	142
C11—H11···O2^ii^	0.95	2.51	3.277 (2)	138
C6—H6···*Cg*1^iii^	0.95	2.88	3.687 (2)	144
C13—H13···*Cg*2^iv^	0.95	2.71	3.519 (9)	143

Symmetry codes: (i) *x*, −*y*−1/2, *z*+1/2; (ii) −*x*+1, −*y*, −*z*; (iii) *x*, −*y*+1/2, *z*+1/2; (iv) −*x*−2, *y*+1/2, −*z*+1/2.
